# Versatile magnetic hydrogel soft capsule microrobots for targeted delivery

**DOI:** 10.1016/j.isci.2023.106727

**Published:** 2023-04-25

**Authors:** Zichen Xu, Zehao Wu, Mingzhe Yuan, Yuanhe Chen, Wei Ge, Qingsong Xu

**Affiliations:** 1 Department of Electromechanical Engineering, Faculty of Science and Technology, University of Macau, Macau, China; 2 Department of Biomedical Sciences and Centre of Reproduction, Development and Aging (CRDA), Faculty of Health Sciences, University of Macau, Macau, China

**Keywords:** Robotics, Magnetic property, Biomedical materials

## Abstract

Maintaining the completeness of cargo and achieving on-demand cargo release during long navigations in complex environments of the internal human body is crucial. Herein, we report a novel design of magnetic hydrogel soft capsule microrobots, which can be physically disintegrated to release microrobot swarms and diverse cargoes with almost no loss. CaCl_2_ solution and magnetic powders are utilized to produce suspension droplets, which are put into sodium alginate solution to generate magnetic hydrogel membranes for enclosing microrobot swarms and cargos. Low-density rotating magnetic fields drive the microrobots. Strong gradient magnetic fields break the mechanical structure of the hydrogel shell to implement on-demand release. Under the guidance of ultrasound imaging, the microrobot is remotely controlled in acidic or alkaline environments, similar to those in the human digestion system. The proposed capsule microrobots provide a promising solution for targeted cargo delivery in the internal human body.

## Highlights

• A novel design of magnetic hydrogel soft capsule microrobots is proposed
• It is physically disintegrated to release microrobot swarms with almost no loss
• The microrobots are remotely controlled for operation in acidic or alkaline liquids
• It provides an innovative strategy for cargo delivery in the internal human body

## Graphical abstract

**Figure:**
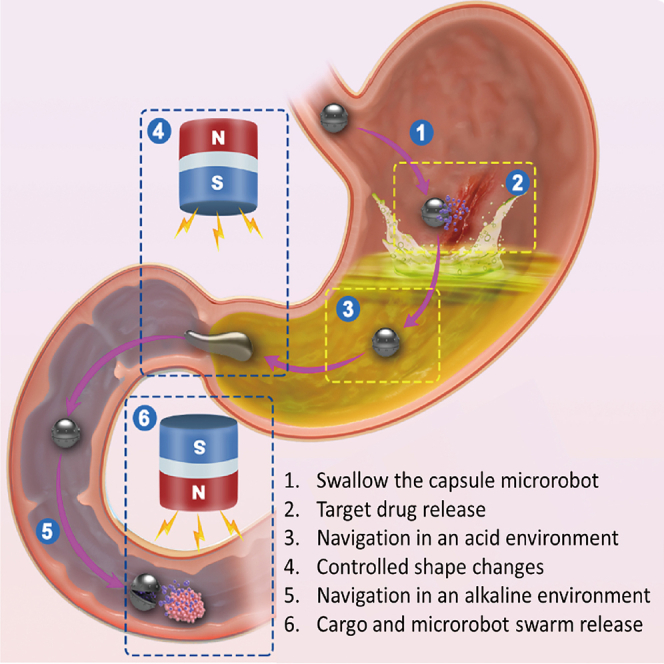

## Introduction

As promising candidates for next-generation biomedical treatments, microrobots have demonstrated attractive advantages in targeted cargo delivery,^1^^,^^2^^,^^3^^,^^4^^,^^5^^,^^6^^,^^7^^,^^8^ medical detection,^9^^,^^10^ and invasive surgeries.^8^^,^^10^ Furthermore, microrobots’ wireless control and actuation enable a deeper exploration of the internal human body, facilitating the extension of current therapies.^8^^,^^11^^,^^12^^,^^13^^,^^14^ In previous research, it is possible for a single microrobot to realize efficient navigation,^8^^,^^11^^,^^15^^,^^16^ actuation,^17^^,^^18^^,^^19^^,^^20^ and control.^21^^,^^22^ However, with limited physical size and volume, enabling an individual microrobot to cope with complex tasks independently is difficult. Currently, an increasing number of microrobots have been devoted to various experiments to improve the performance of single microrobots.^1^^,^^23^^,^^24^^,^^25^^,^^26^^,^^27^^,^^28^ Corresponding microrobot swarm control methods play a profound role in efficiently organizing microrobot groups.^28^^,^^29^^,^^30^^,^^31^^,^^32^ Notwithstanding splendid achievements in microrobot swarm pattern generation,^29^^,^^30^^,^^33^^,^^34^ navigation,^32^^,^^35^ and manipulation,^24^^,^^36^ guaranteeing the stability of microrobot swarm systems in front of a complex flowing environment remains a difficult problem, especially during operations within the human body.

Microrobot swarms induced by global field-based actuation tend to form weak connections among micro individuals, impeding the ability to retain all individuals in the swarms within fluidic environments.^1^^,^^24^^,^^25^^,^^26^^,^^27^ During long navigations within vascular networks or digestive systems, the lost microrobot individuals may reside in the human circulatory system, leading to potential danger. In addition, many experiments have been conducted in ideal setups where the operation substrates are relatively smooth, such as Petri dishes and glass tubes.^24^^,^^25^^,^^26^^,^^27^^,^^33^^,^^34^ Under such a perfect experimental setup, there is always an inevitable loss of microrobot individuals. When navigating the surface of human body tissues, uneven substrates and accompanying mucus might lead to worse results. This shortcoming not only reduces the utilization of the microrobot swarm but also makes the recycling procedures more difficult. Individual microrobots can be easily lost in the environment, and it is even tougher to ensure the integrity of the carried cargo. Therefore, developing a reliable method to realize long-range delivery of microrobot swarms and other cargoes without individual loss is desirable.

Inspired by the traditional drug release method, medical capsule designs might be a potential solution to greatly reduce drug waste during transportation in the human circulatory system. Capsule disintegration and drug release impose high demands on the environment and time. For traditional oral capsules, precisely and remotely controlling the drug release process remains challenging. To solve this problem, materials and responsive mechanisms are the key factors to be considered. Hydrogel-based biocompatible materials are increasingly attractive due to their considerable biocompatibility and potential multifunctionality.^37^^,^^38^^,^^39^^,^^40^^,^^41^ Responsive disintegration of hydrogel materials provides a good candidate for capsule structure designs, enabling controllable release by magnetic response,^39^^,^^40^^,^^42^^,^^43^ biological reaction response,^44^ pH response,^45^^,^^46^^,^^47^ and light-heat response^48^^,^^49^ (Table 1). For *in vivo* operations in deeper tissues, magnetic hydrogel materials are mainly utilized for actuation and delivery,^39^^,^^40^^,^^42^^,^^43^ while light penetration depth severely limits the light-heat response mechanisms.^42^^,^^48^ The relatively stable human internal environments cannot always fulfill the requirements for the pH and biological reaction response mechanisms.^44^^,^^45^^,^^46^^,^^47^ Thus, a new on-demand disintegration mechanism should be developed to produce more practical hydrogel-based capsule structures.

**Table 1:** Comparison of responsive hydrogel-based designs for on-demand release

Hydrogel	Actuation	Triggering agent	Wireless control distance	Reference
Metallo-alginate	Unable	Microwave (2.45 GHz)	None	Zhu et al.^50^
Gelatin/PVA	Magnetic field	Near-infrared light	<1 cm	Kim et al.^42^
Gelatin methacrylate	Magnetic field	Enzymatic biodegradation	None	Noh et al.^44^
Polyethylene glycol	Unable	pH/Glucose	None	Yesilyurt et al.^46^
Agarose	Unable	Near-infrared light	<1 cm	Qiu et al.^49^
Calcium alginate	Magnetic field	**Gradient magnetic field**	**≈3 cm**	This work

**Figure 1:**
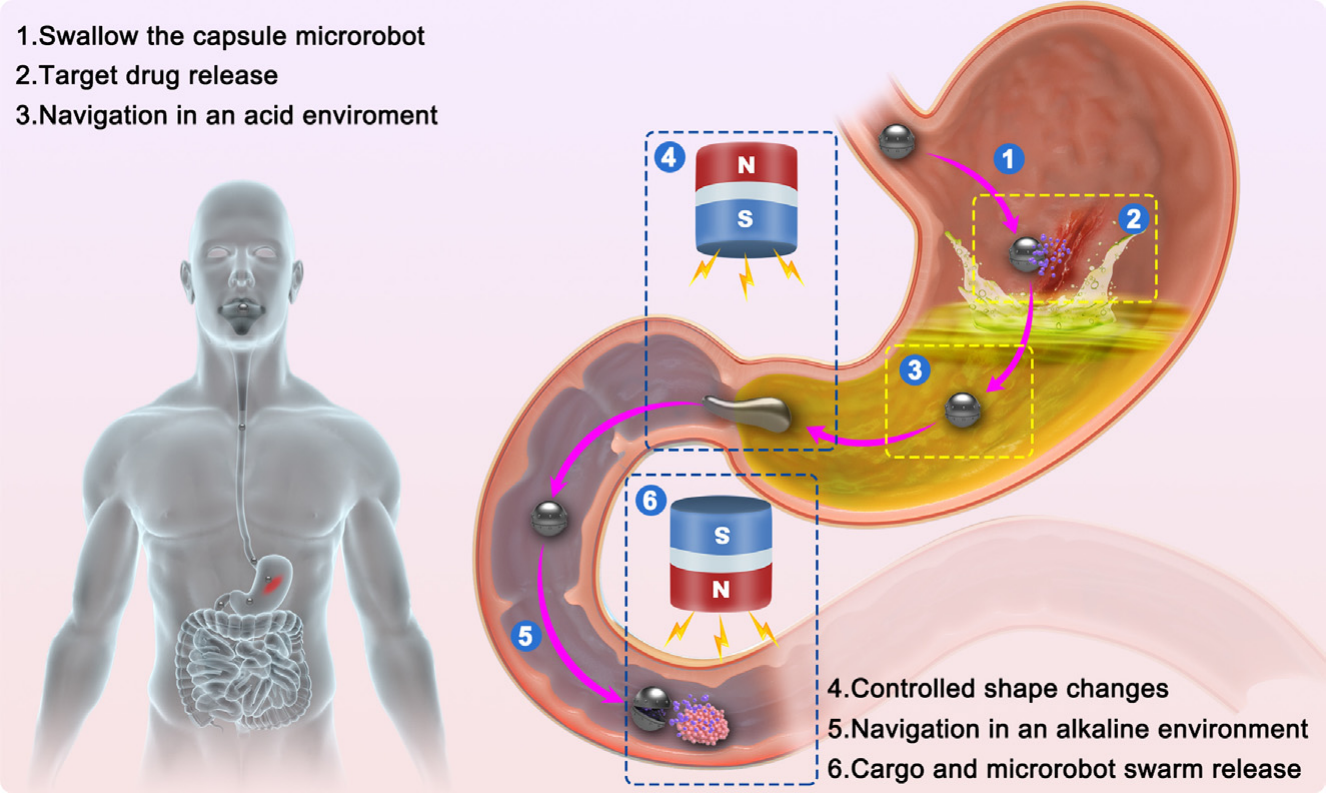
Schematic of versatile hydrogel soft capsule microrobot execution in the human digestion system

In this paper, we propose a novel approach by introducing a pure physical disintegration mechanism for the on-demand release of magnetic hydrogel capsule microrobots (Figure 1). Inspired by the embryo’s structure, the microrobot is designed based on bionic factors, where a thin hydrogel membrane encloses microrobot swarms. The disintegration procedure is conducted from the inside out, which is dominated by the magnetic field-induced physical interactions among those microparticles. Strong gradient magnetic fields condense all magnetic microparticles together, including the microparticles left inside the hydrogel membrane. Hence, physical triggers can entirely break hydrogel-based mechanical structures to release the cargo on demand. In addition, the physical force interaction triggering mechanism can avoid causing potential damage to the enclosed cargo’s biological or chemical properties. The natural sedimentation method is introduced to generate suspension droplets (composed of magnetic powder and CaCl_2_ solution) for mass production of this novel microrobot. The droplets are produced with almost the same physical parameters. Hydrogel magnetic soft capsule microrobots are generated by adding those generated droplets into sodium alginate solution. The thickness of the hydrogel membrane can be controlled by adjusting the concentrations of CaCl_2_ solution and sodium alginate solution. The reaction time also plays an essential role in this formation procedure. Moreover, the shape of capsules can be custom-designed for more complex tasks by 3D printing technologies. The magnetic field-based actuation mechanism is theoretically proven by conducting physical analysis and COMSOL simulations, which are verified through experimental studies. Extensive experiments show that it can deliver various cargoes, including drug solutions, micro-objects, and live organisms like zebrafish embryos. The microrobot can maintain its entire structure in acidic (pH 1.5) or alkaline (pH 12) environments. Even in front of narrow channels and rugged surfaces, there is almost no individual loss during navigation. Due to the adoption of biocompatible materials, 90% of zebrafish embryos can develop normally, even having been enclosed in the capsule microrobot for 24 h. Using ultrasound imaging equipment allows for monitoring real-time navigation in an opaque environment. In deeper tissues, the on-demand release procedure can be realized where the wireless control distance is longer than existing research work (Table 1). In fact, with the help of well-designed external magnetic actuation equipment, the distance can still be further promoted to affect deeper tissues. This novel design has been proposed to ensure efficient, diverse mass cargo delivery and reduced cargo loss.

## Results

### Fabrication of magnetic soft capsule microrobots

**Figure 2:**
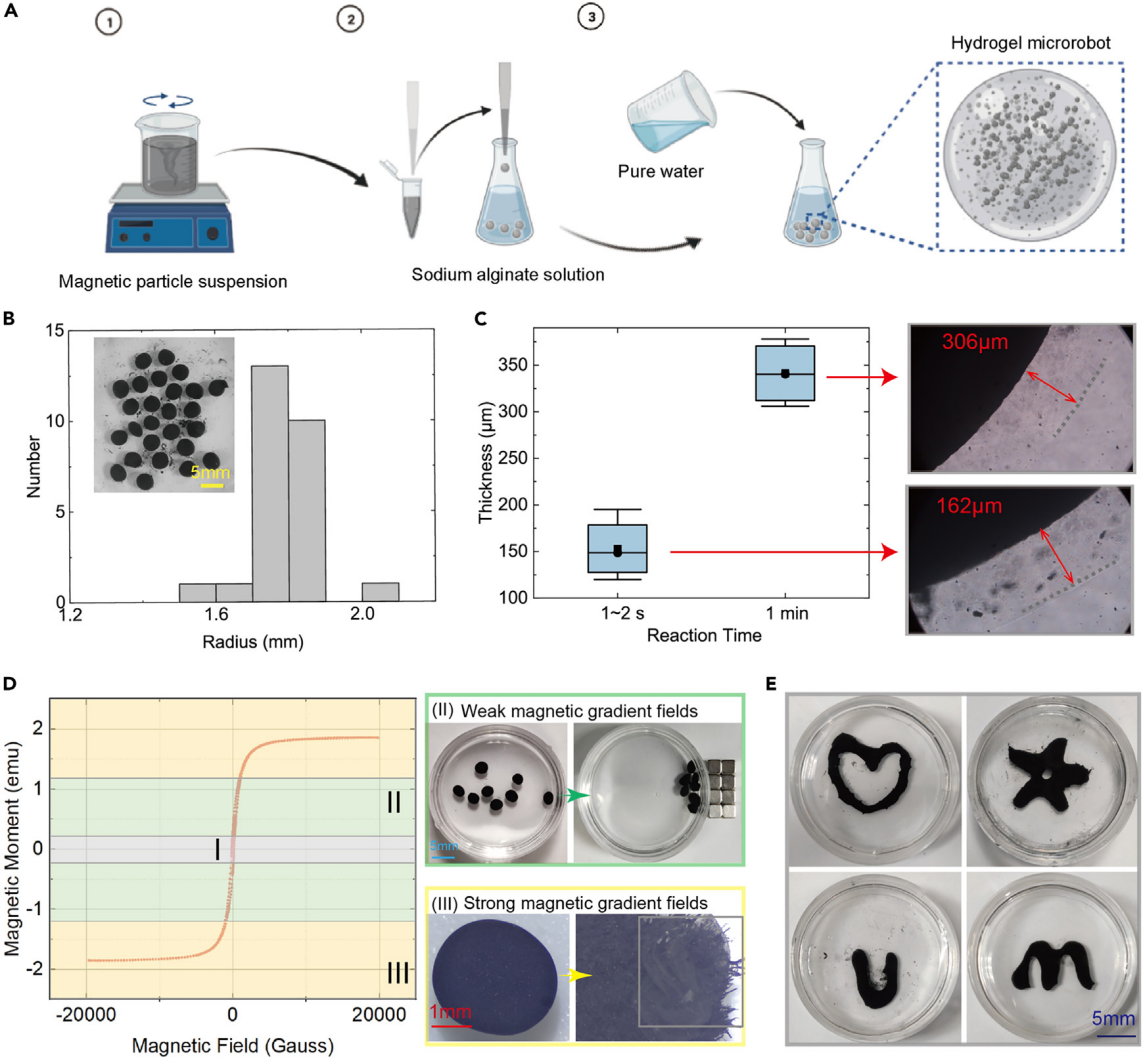
Fabrication of magnetic hydrogel soft capsule microrobots (A) Schematic image of the procedure for mass production of the microrobots. (1) A partial amount of the CaCl_2_ solution and the magnetic powder was added to produce a suspension. (2) Addition of suspension droplets into sodium alginate solution. (3) Pure water was added to dilute the sodium alginate solution. (B) Produced hydrogel microrobots’ size distribution. (C) Thickness of microrobots that are produced with different reaction times. The insets indicate the experimental images. (D) Actuation analysis under different magnetic fields. The curve is the magnetic hysteresis loop of Fe_3_O_4_ microparticles. The area I is the dead area, where the magnetic microrobot cannot be actuated. In area II, the microrobot can be well-actuated without damaging its physical structures. It is the response to weak gradient magnetic fields, where the microrobots converge together with no breakage of mechanical structures. In area III, it is the response to strong gradient magnetic fields, where the microrobot’s mechanical structures are broken with a strong interaction force applied. The images indicate that the microrobot is composed of a thin hydrogel membrane and magnetic microparticles. Inside the gray box, the broken structure of the hydrogel membrane is clearly shown. The divide among those areas depends on the utilized materials and fabrication procedures. (E) Specially designed microrobots fabricated by 3D printing.

Magnetically responsive and controllable microrobots were fabricated based on suspension droplet generation. Using the natural sedimentation method, suspension droplets composed of CaCl_2_ solution and magnetic microparticles were produced with the desired sizes. By adding those droplets to the alginate sodium solution, hydrogel membranes can be formed to enclose those droplets (Figure 2A) due to relevant chemical reactions. The whole procedure is pretty easy, and it can be finished by using pipettes to produce plenty of relatively similar microrobots (Figure 2B). We aim to create hydrogel membranes with proper mechanical strength for capsule microrobot design. This feature means the membrane is strong enough to resist complex external interactions during navigation in the human body’s internal environments. On the other hand, the membrane should also be weak enough to be disintegrated by the applied interactions induced by external magnetic fields. Thus, the thickness of the membranes is the critical factor to be considered. To avoid generating overly thick membranes, the suspension droplets stayed in the alginate sodium solution for only 1–2 s. Then, they were taken out of the solution quickly and transferred to pure water to prevent further chemical reactions. Longer reaction time will contribute to thicker hydrogel shells (Figure 2C). The thickness of the generated hydrogel membranes is governed by the combined effect of CaCl_2_ solution concentration, alginate sodium solution concentration, and reaction time of the droplets staying in the alginate sodium solution. Optimal designs of soft magnetic capsule microrobots can be performed to cater to the different requirements of diverse tasks.

The introduction of magnetic microparticles plays a significant role in this work. It enables efficient magnetic actuation and acts as the basis of the magnetic field-based disintegration mechanism (Figure S1). During magnetic actuation, the actuated microparticles can be divided into microparticles embedded in hydrogel membranes and microparticles enclosed by hydrogel membranes. In the former case, when some magnetic microparticles are actuated, their movement will cause crevices in the hydrogel shell and destroy it. In the latter case, when the microparticles are organized into powerful microrobot swarms inside the capsules, the accompanying physical interactions will apply pressure and destroy the completeness of the membranes. These two situations play dominant roles in the breaking mechanism of the capsules. To reveal the feasibility of the property, several hydrogel microrobots were produced, where we mixed 5.0 g Fe_3_O_4_ particles (<5 μm) and 2.5 g CaCl_2_ solution (1% concentration) to generate the desired suspension. Suspension droplets were added to the alginate sodium solution (0.5% concentration) using a dropper. The capsule microrobots can remain stable for over three days in pure water. In actual applications, by applying a relatively weak (≈3 T/m) and stable magnetic gradient field using eight small permanent magnets (NdFeB, dimension: 4 mm × 4 mm × 4 mm), the capsule microrobots are gathered with several irregular shape changes (Figure 2D). Under such circumstances, the capsule microrobots can be actuated to generate flexible motions without structural damage. Furthermore, the enclosed materials are secure during actuation, enabling long-range cargo delivery. For on-demand cargo release, we introduced strong (>6 T/m) magnetic gradient fields to destroy the capsule structure (Figure 2D). In principle, the magnetized object suffers from a magnetic force in the magnetic fields. The magnetic force is theoretically calculated as follows,

(Equation 1) $$F_{m}=\int_{V_{m}}\left(M\cdot \nabla \right)B\;dV_{m}$$

where V_m_, M, and B denote the volume of the magnetized object, the magnetization of the object, and the flux density of the magnetic field, respectively. It helps to calculate the induced magnetic forces to prevent potential physical injuries within the human body. A large cylindrical NdFeB permanent magnet (diameter: 30 mm, height: 30 mm) was utilized to supply strong physical interactions. By moving the magnet, a changing magnetic field is produced, which will intensify the physical breaking effects caused by those interaction forces. Finally, the hydrogel membranes are destroyed, and the enclosed cargoes are released. For larger particles, the destruction procedure can be observed more clearly (Figure S2; Video S1). The magnetic microrobot swarm can also be easily recycled (Video S2). The magnetic hysteresis loop of utilized Fe_3_O_4_ particles was measured by a vibrating sample magnetometer. The weight of the measured particles is 24.5 mg. In addition, the manufacturing procedure can be realized through 3D printing technology. Several personalized structure microrobots are produced using a dropper to continuously inject the suspension into the alginate sodium solution (Figure 2E). This way, more bioinspired functions can be integrated into the capsule microrobot design. This work provides a novel solution for wirelessly controlled release.

Video S1. Navigation of the capsule microrobot and release of microrobot swarm, Related to Figure 2

Video S2. Recycle procedure of released microrobot swarm, Related to Figure 2

### Soft structures and adaptive behaviors of the capsule microrobot

**Figure 3:**
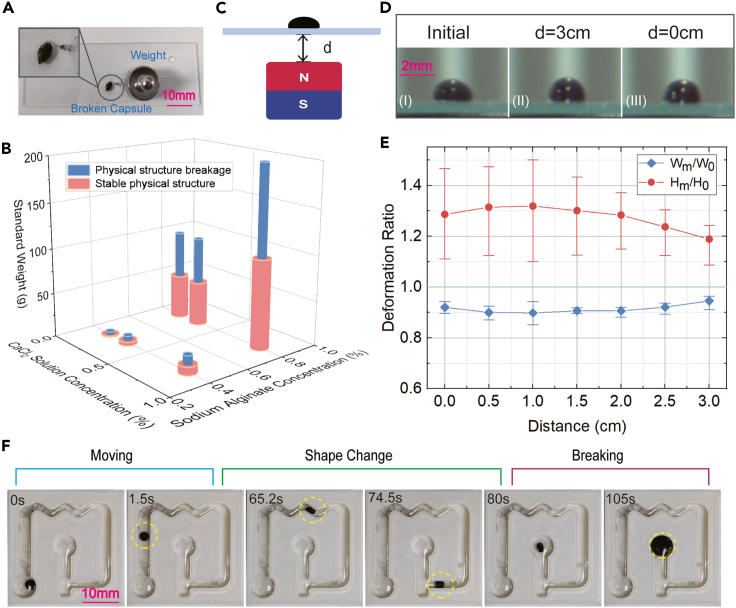
Testing and application of the soft property of the microrobots (A) Image of a capsule microrobot that was crushed. The capsule structure is destroyed by putting a heavy enough weight on the capsule microrobot. (B) Experimental results of the microrobots’ ability to withstand stress. (C) Schematic of the gradient magnetic field-induced shape change. (D) Images of the experimental results of shape change. (E) Experimental results indicate the different degrees of deformation caused by gradient magnetic fields. The distance is *d,* as presented in (C). The deformation ratio indicates the ratio of the deformed width or height to the initial width or height. (F) Navigation of a capsule microrobot in complex channels by utilizing the property of flexible deformation.

The hydrogel shells’ mechanical properties are significant to resist environmental interactions and protect the enclosed cargo. The force in one direction is hard to wreck the hydrogel membrane structures. This property guarantees the stability of capsule microrobots during navigation, where common collisions cannot disintegrate the capsule structures. We produced diverse capsule microrobots and tested their performances further to reveal the relationship between mechanical strength and manufacturing details. We used standard weights to squash the hydrogel microrobots to present the ability to resist external forces (Figure 3A). The solutions of CaCl_2_ and alginate sodium with different concentrations reacted to generate hydrogel membranes with different physical properties, where the reaction time was kept the same. The hydrogel shells produced by high-concentration solutions are more durable. It enables microrobots to resist stronger external applied forces (Figure 3B). These results are valuable for producing personalized capsule microrobots aimed at designated tasks.

The hydrogel structures are soft, contributing to flexible deformations and avoiding rigid collisions. To present its soft property, we adjusted the distance between a permanent magnet (diameter: 30 mm, height: 30 mm) and a capsule microrobot so that different gradient magnetic fields are generated and the applied magnetic forces are controlled (Figure 3C). Then, we observed and recorded the deformation of the capsule microrobots (Figure 3D). The unchanged gradient magnetic fields provide constant interaction forces, where the capsule microrobots can remain stable for at least 1 h. Inside the capsule, the content can be regarded as magnetic fluid, subject to external magnetic fields. However, limited by the hydrogel membrane shell, their shape changes are not arbitrary. Experimental results show that the capsule becomes sharp as the gradient magnetic field strength increases. When the gradient is strong enough, its height will be reduced. The width of the capsule microrobot follows the opposite tendency of variation (Figure 3E). The related physical interaction will be changed by tuning the magnetic force direction. Intuitively, this procedure can be regarded as pinching plasticine. In a crowded channel, the deformation property enables efficient passage (Figure 3F; Video S3). Based on these experimental results, there is a clear and large enough dividing area between the situations of deformation and disintegration, which makes it possible to use a single actuation method to realize the two modes, i.e., actuation and release.

Video S3. Capsule microrobot’s deformation to navigate in a crowded channel, Related to Figure 3

### Magnetic actuation and wireless controlled release of capsule microrobots

**Figure 4:**
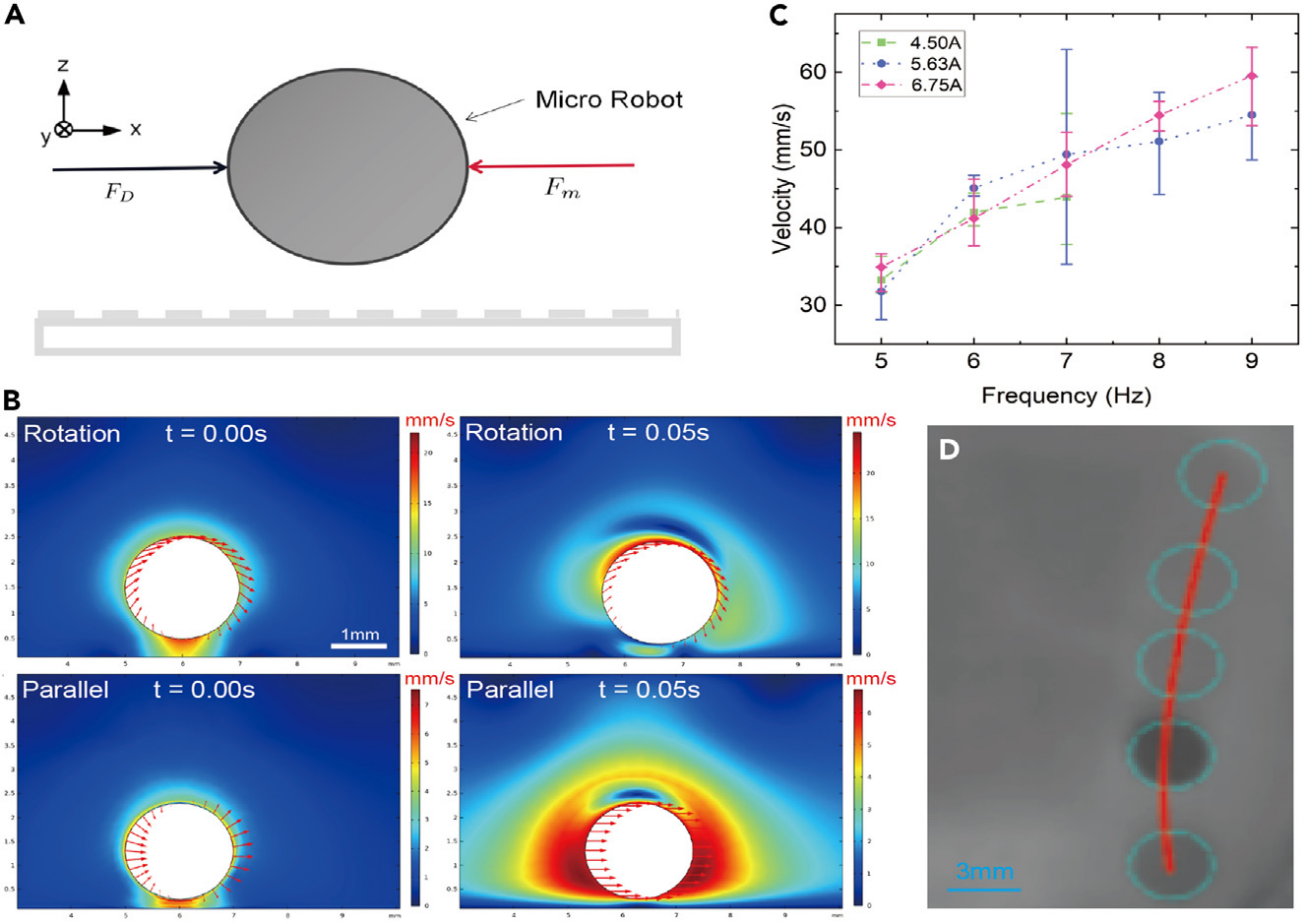
Analysis results of actuation for the capsule microrobot (A) The parallel motion generation force diagram by a gradient magnetic field. (B) Simulation results of the induced flow velocity distribution obtained with COMSOL, including the rotation and parallel movements. (C) Experimental results of rotating magnetic field actuation. (D) Images of trajectory results of a capsule microrobot.

The introduction of magnetic materials enables efficient magnetic actuation. Magnetic force-based and magnetic torque-based control are the main actuation methods for wireless control of magnetic microrobots. The magnetic forces drive the microrobot in the direction of forces, and magnetic torques rotate the microrobot to move forward by rolling. Given the capsule microrobot’s physical size, several force interactions dominated at a small scale, such as the capillary effect, van der Waals interactions, and electrostatic charging, can be ignored. Strong magnetic forces and torques serve as the main governing factors. As illustrated in Figure 4A, *F*_*m*_ denotes the magnetic force and *F*_*D*_ is the fluidic interaction force. Hydrodynamics resistance is the critical effect of the motion of microrobots. We introduced the drag force equation to approximate the interactions generated by fluid, i.e., the viscous drag force *F*_*D*_, as follows.

(Equation 2) $$F_{D}\approx \frac{1}{2}\eta \rho Sv{\;}^{2}$$

where *η* denotes the drag coefficient, *ρ* is the density of the fluid medium, *S* is the contact area facing the flowing fluid, and *v* is the velocity of the liquid. By approximating the induced fluidic interaction forces, it is essential to guarantee the completeness of the capsule microrobot by selecting a proper actuation strategy to adjust the distribution of the induced flow velocity. Simulations were conducted with COMSOL software to reveal the distribution of the induced flow velocity. The rotation movement of microrobots generates a smaller influenced flow area in the initial static environment (Figure 4B). Through experimental tests, we found that weak rotation magnetic fields (<10 mT) with little gradients can still actuate the microrobots. Using permanent magnets for microrobot actuation is easier to implement in practice. Nevertheless, powerful gradient magnetic fields will crush the capsule, which needs to be carefully considered. Compared with the promotion of magnetic field density, the rise of rotation frequency performs a more significant role in speeding up the microrobots (Figure 4C). During the manufacturing procedure, the shape of microrobots is not a perfect sphere, which affects the actual movement. Their rotation is not so fluent, and there are specific errors in their motion trajectory control. As a result, the actual trajectory is a curve rather than a planned straight line (Figure 4D). In different fluidic environments, the flexible selection of an actuation strategy is vital for motion generation.

**Figure 5:**
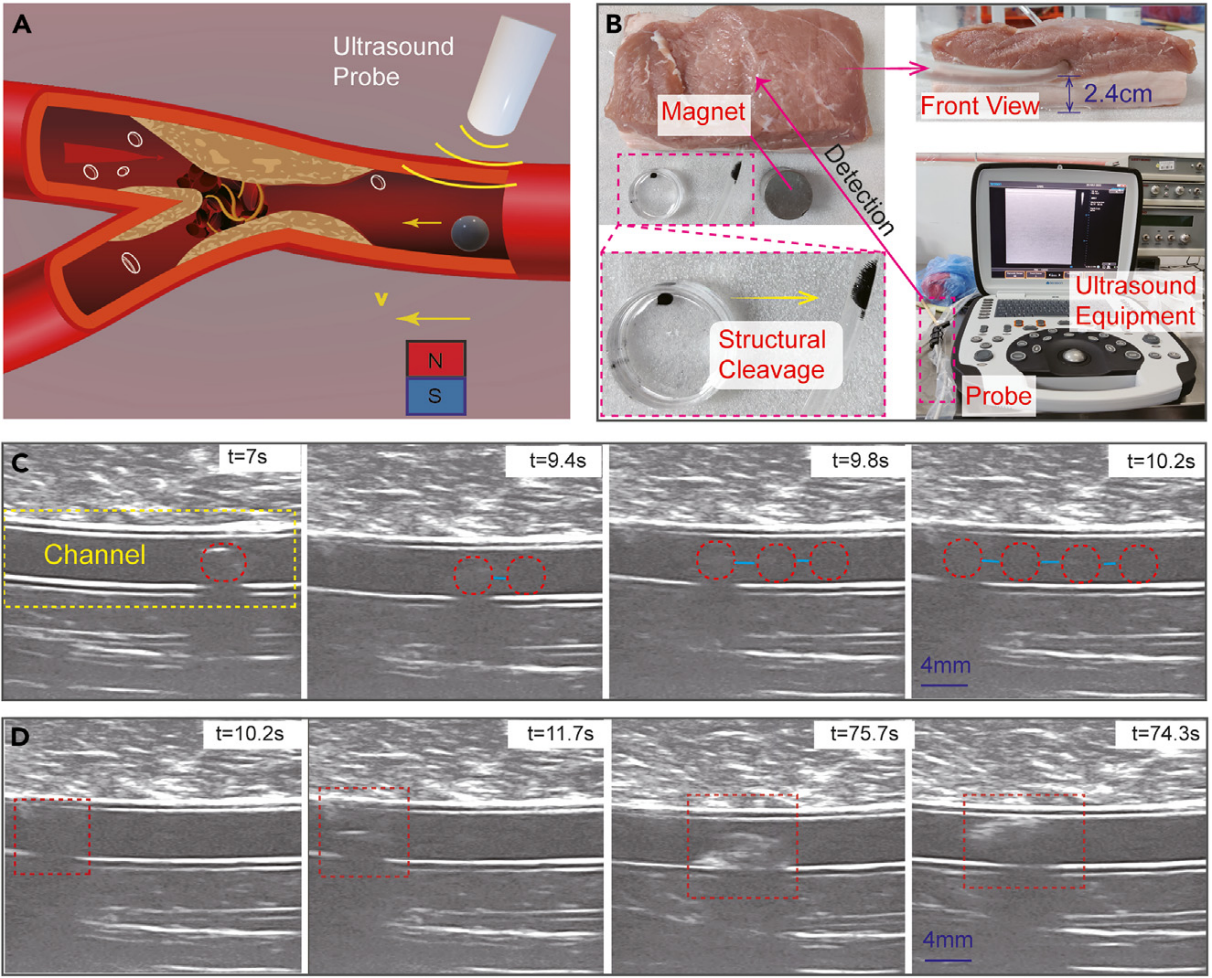
Controlled navigation and disintegration of capsule microrobots under the guidance of ultrasound imaging equipment (A) Schematic of a capsule microrobot’s navigation in the vessel, which is guided by ultrasound imaging equipment. (B) Experimental platform setup. (C) Ultrasound images during the capsule microrobot’s navigation actuated by a permanent magnet. (D) Ultrasound images during capsule microrobot disintegration.

Remote-controlled release remains a challenge for many cargo delivery tasks in the human body’s internal environment. Many wireless responsive mechanisms rely on chemical or biological environments, and several physical stimuli cannot reach deep tissues. In previous research, magnetic fields have played an important role in remote actuation and control. Introducing hydrogel membranes with controllable thickness for well-designed capsule microrobots provides a new wireless response mechanism. Under gradient magnetic fields, the induced interactions facilitate the disintegration of capsule structures over a relatively long distance (2–3 cm), which performs better than the current near-infrared photothermal trigger mechanisms (<1 cm). For actual medical scenarios, ultrasound imaging equipment is utilized for real-time image feedback (Figure 5A). We used a piece of pork to mimic the human body tissue. A plastic tube (internal diameter: 3.8 mm) served as an artificial vessel placed inside the pork approximately 2.4 cm from the tissue surface (Figure 5B). Through observation by ultrasound imaging equipment, the capsule microrobot’s position was obtained in real time. A permanent NdFeB magnet (diameter: 30 mm, height: 30 mm) provided gradient magnetic fields to actuate the microrobot in the desired direction. To protect the completeness of capsule structures during navigation, the magnet moved in a single direction without change (Figure 5C). When the capsule microrobot arrived at the designated position, we rapidly rotated (with the frequency of 1–2 Hz) and moved the magnet to intensify the induced physical interaction. Part of the magnetic microparticles was stripped from the hydrogel shell, and the enclosed microparticles were organized into a powerful microrobot swarm, which disrupted the mechanical structures. Dominated by those interactions, the enclosed contents were successfully released (Figure 5D; Video S4). Continuously intensifying the physical interactions between microrobots and environments contributes to the changes in hydrogel shells’ mechanical structures, which provides a novel reference for designing next-generation microrobot release mechanisms.

Video S4. Navigation of the capsule microrobot and release microrobot swarm under the guidance of ultrasound-imaging equipment, Related to Figure 5

### Potential application of the capsule microrobots

**Figure 6:**
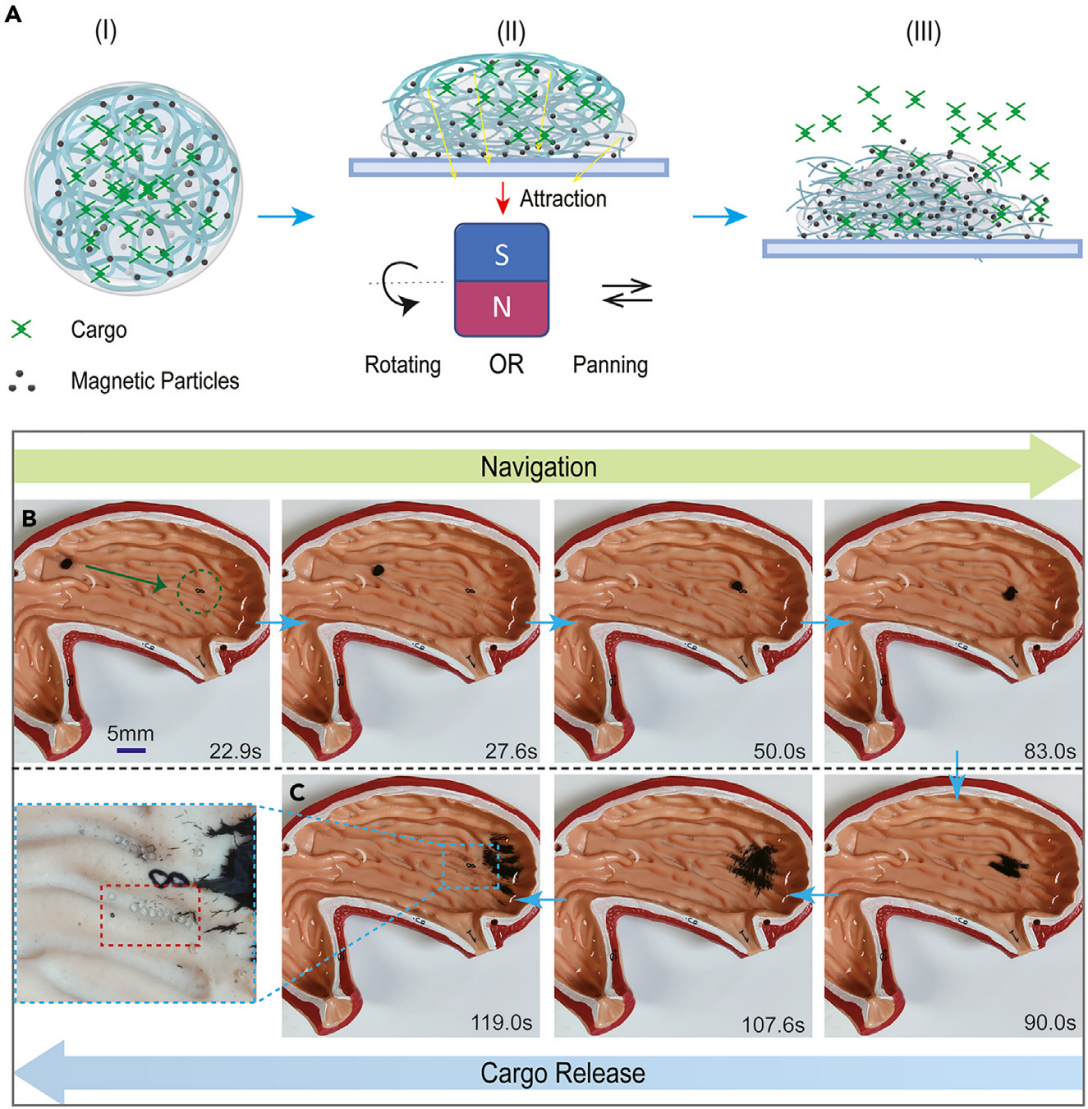
Micro-object delivery by the capsule microrobot (A) Schematic of the disintegration procedure of the capsule microrobot. (B and C) Micro-object delivery and controlled release by the capsule microrobot on a 3D rugged surface.

Loading capacity is vital for microrobots to execute cargo delivery tasks. The capsule’s mechanical structure enables diverse cargo loading and delivery in this work. In addition, the outstanding biocompatibility of hydrogel-based materials fulfills the requirements of potential biomedical applications. Given the manufacturing details and generation procedure of the hydrogel membranes, we propose adding several micro-objects to the magnetic microparticle suspension. The suspension is dense and is produced from CaCl_2_ solution and magnetic powder at a mass ratio of 1:2. Hence, the micro-objects can be evenly distributed after stirring. This procedure guarantees that the micro-objects are successfully enclosed in the suspension droplets. Then, the generated capsule microrobots realize micro-object loading. In extreme environments within the internal human body, such as the digestive system, ensuring that the microrobot remains stable in acidic or alkaline environments is essential. Experiments were conducted to demonstrate such properties. The hydrogel capsule microrobot can maintain its entire structure in an acidic environment (pH 1.5) for more than 2 h and in an alkaline environment (pH 12) for two days (Figure S3; Video S5). We used a permanent magnet to supply evolving gradient magnetic fields to release the enclosed cargos, intensifying the induced physical interactions to disrupt the hydrogel-based mechanical structures (Figure 6A). We utilized glass microballs (≈800 μm) as the desired cargos to further reveal the effectiveness, where several glass microballs were enclosed in the capsule microrobots. Under the external actuation of a NdFeB permanent magnet, the capsule microrobot succeeded in carrying the enclosed cargos and navigating on a 3D uneven surface. When the microrobot arrived at the designated position, we applied the changed gradient magnetic fields by rapidly moving the permanent magnet. Then, the hydrogel membrane was disrupted, and the glass microballs and magnetic microparticles were successfully released (Figures 6B and 6C; Video S6). During navigation, there is no loss of cargo or magnetic microparticles, which significantly promotes delivery efficiency.

Video S5. Capsule microrobot movement in an acidic environment, Related to Figure 6

Video S6. Navigating the capsule microrobot and releasing microobjects on a 3-D uneven surface, Related to Figure 6

**Figure 7:**
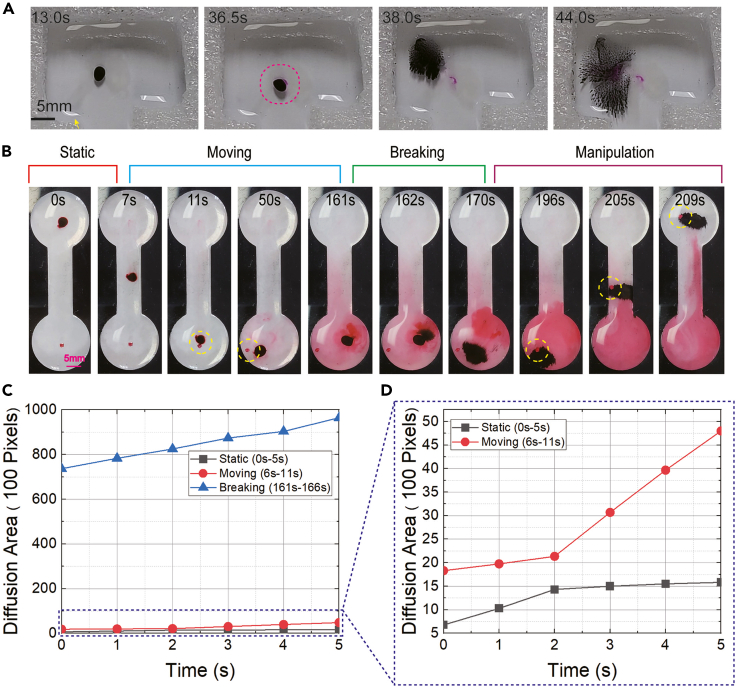
Controlled drug release, diffusion, and microrobot swarm manipulation (A) Drug solution delivery and controlled release in the contact area. (B) Experimental images indicate the procedure of microrobot swarm release, induced controlled drug diffusion, and swarm-based manipulation. In the desired place, the microrobot swarm can efficiently grasp and carry the micro-object. (C) The drug diffusion area and time relationship in different steps, including static, moving, and breaking situations. The drug diffusion area increased as time went on. (D) Comparison of the diffusion velocities of the static and moving situations.

The delivery capability of the microrobots for several special cargos was also verified. Within the internal human body, the water-based environment is the main scenario for biomedical applications. In addition, many drugs work in solution form. Therefore, transporting drug solutions for *in vivo* treatment is valuable. Here, alginate sodium hydrogel serves as an artificial lesion tissue made from phenolphthalein solution, CaCl_2_ solution, and alginate sodium hydrogel solution. As designated drugs, the Ca(OH)_2_ solution replaces a part of the CaCl_2_ solution to produce capsule microrobots. Phenolphthalein is the indicator used to visualize OH^−^ diffusion, namely, drug diffusion. Given the properties of hydrogel membranes, it is possible to slowly release the drugs without mechanical structure breakage. Therefore, we can disrupt the capsule structure and ultimately release the drug for a more efficient release procedure. Furthermore, during the changing magnetic fields, the released magnetic microparticles are organized into tentacle-like microrobot swarms, accelerating drug diffusion (Figure 7A; Video S7). To further demonstrate the properties, we utilized red ink as the desired drug solution, enclosed in the capsule microrobot (Figure 7B). We calculated the red area to describe the drug release by counting the corresponding pixels. The drug release procedure is divided into three steps, including the static situation, the moving situation, and the breaking situation. We recorded the data during the initial 5 s of every step and compared their velocity (Figures 7C and 7D). It is obvious that the diffusion was speeded up when moving, and the diffusion was extremely fast when breaking. What’s more, the released microrobot swarm can execute assignments in the desired position, proving the effectiveness of microrobot swarm transportation and its versatility (Figure 7B; Video S9).

Video S7. Drug solution delivery and release of the capsule microrobot, Related to Figure 7

Video S9. Controlled drug and microrobot swarm release and related microrobot swarm manipulation, Related to Figure 7

**Figure 8:**
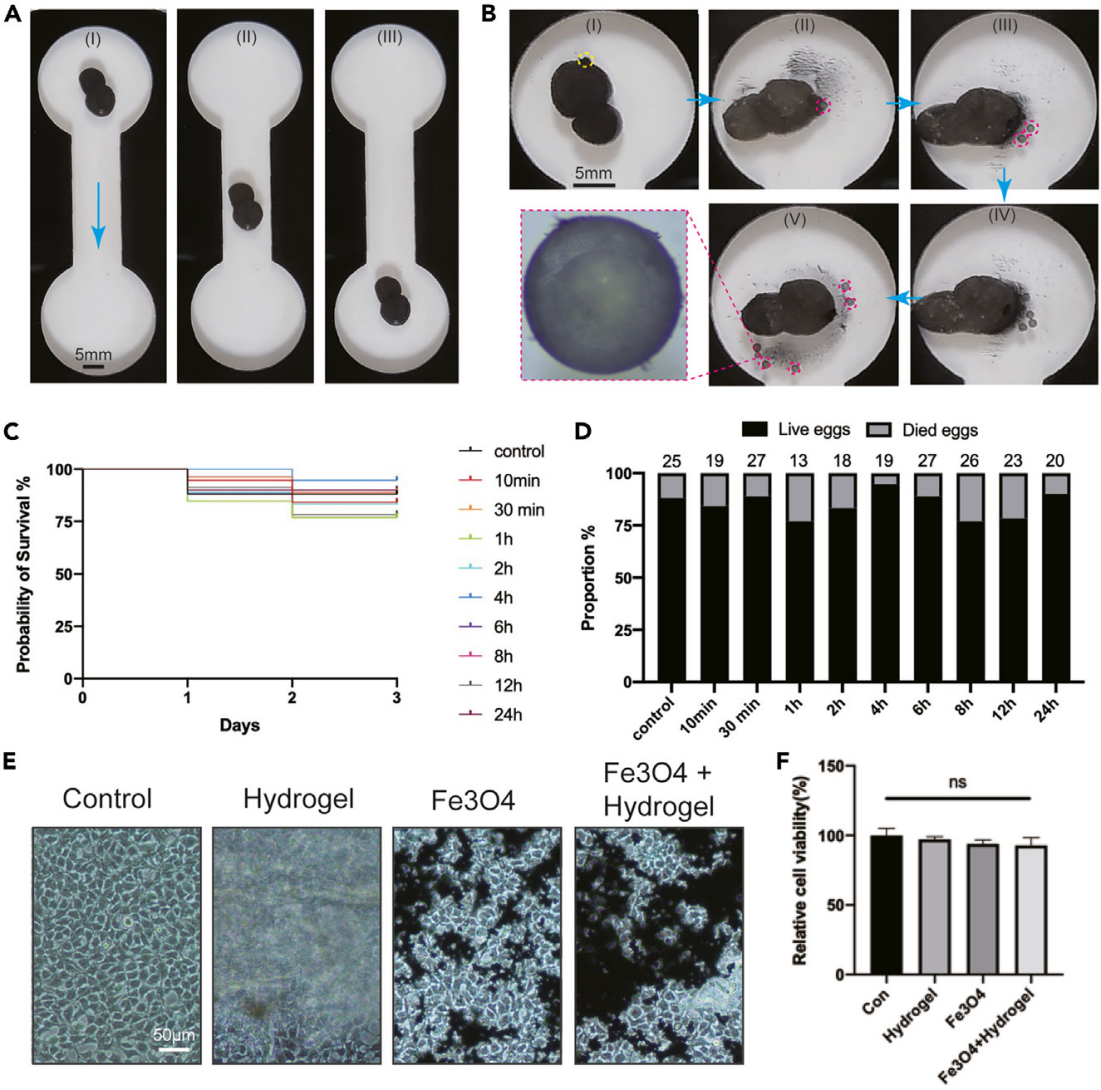
Zebrafish embryo delivery and controlled release, and biocompatibility evaluation (A and B) Zebrafish embryo delivery and controlled release. (C) The probability of survival curves of ten test groups. (D) Final survival proportion of all groups of zebrafish embryos. There is no statistically detected difference between the groups in this experiment (p > 0.05). (E) Experimental images of cell toxicity testing. (F) Relative cell viability after co-culturing with related materials.

Due to the considerable biocompatibility of utilized materials, they can be used to carry live samples for biological applications. Taking zebrafish embryos as examples, we produced capsule microrobots containing several embryos, which might be a valuable reference for related artificial insemination operations. Here, to protect the embryos, we utilized a low concentration of CaCl_2_ solution (0.5%) to produce the suspension. The thickness distribution of hydrogel membranes is modified by tuning the contact area between the droplets and the alginate sodium solution. This operation provides several new designs for the novel capsule microrobot. To precisely release the embryos, we design the microrobot into gourd shapes with only one outlet. Magnetic fields control the expansion and contraction of the gourd capsule microrobot. Then, the embryos can be released one by one (Figures 8A and 8B; Video S8). We enclosed many zebrafish embryos inside the hydrogel capsules to further reveal the excellent biocompatibility of the proposed microrobots. After holding zebrafish embryos in the capsule microrobots for different timelines (e.g., 10 min, 30 min, 1 h, 2 h, 4 h, 6 h, 8 h, 12 h, and 24 h), we released them and cultured them in a typical environment (Figure S4, Supplementary Information). By recording the final survival of embryos, we examined the effects caused by enclosing embryos in microrobots (Figure 8C). Even after holding the embryos in the magnetic hydrogel capsules for 24 h, 90% of embryos successfully developed normally. The final survival rates of the nine experimental groups were approximately 80% (Figure 8D). The testing and related statistical analysis results indicate no statistically detected difference between the experimental groups (p > 0.05). HEK293 cells were also used to further evaluate the *in vitro* biocompatibility of the Fe3O4, hydrogel, and the mixture of hydrogel and Fe3O4 suspension (Figure 8E). After co-culture of the HEK293 cells with the experimental materials, the MTT assay was used to analyze the cell proliferation and viability. The results showed no significant difference in cell viability among control and treatment groups (Figure 8F, p > 0.05), which confirmed our *in vivo* data in zebrafish embryos.

Video S8. Live zebrafish embryo delivery and precise release via the capsule microrobot, Related to Figure 8

## Discussion

This paper reports a novel magnetic soft capsule microrobot design for long-distance on-demand release, where hydrogel-based structures can be entirely disrupted by applying external magnetic fields. This wireless disintegration mechanism eliminates the dependence on special environmental conditions, such as pH value and biological enzymes. Compared with photothermal degradation, the magnetic field performs better in penetrating deeper than near-infrared (<1 cm). Current photothermal-responsive designs are mainly applied in superficial disease treatment. In our experiments, the maximum control depth under the tissue was approximately 3 cm, where the capsule microrobot was efficiently actuated and successfully disintegrated. This design introduces the responsive disintegration mechanism based on gradient magnetic fields, providing a new reference for hydrogel-based responsive mechanism designs. More intriguingly, the capsule microrobots are soft, which allows better contact with lesions by avoiding physical collision injury. The accompanying deformation ability enables the microrobots to execute tasks in a more complex environment, such as navigating in a channel with physical sizes smaller than the microrobots. Based on the reported novel design, similar hydrogel materials can also perform such functionalities.

Capsule microrobots have attractive advantages in carrying diverse cargos, ranging from micro-objects to drug solutions. Due to the usage of biocompatible materials, the delivery procedure is nontoxic and harmless to zebrafish embryos. In theory, if the micro-objects can remain stable in a relatively high Ca^2+^ solution (0.16%–1.00%) after adding the desired micro-objects to the magnetic powder suspension to produce droplets, numerous types of cargo can be enclosed in the capsule and delivered to the desired location. This property will facilitate various tasks of targeted delivery. In addition, in extreme environments, such as acidic (pH 1.5) or alkaline (pH 12) environments, the microrobot can remain stable for a long time (>2 h). We successfully transported zebrafish embryos to the desired location for potential artificial insemination operations. The embryo release procedure was precisely controlled, after which the released embryos stayed alive and developed as expected. Because of the intense physical interactions within the capsule, the enclosed cargo should be protected in advance, or the induced interactions will damage the cargo. The gradient magnetic fields help to destroy the hydrogel membrane, where the magnetic forces might influence the physical situation of the enclosed cargos, such as stem cells or micro-components. This scenario is the main limitation of this novel design. The capsule structure brings a new solution for targeted delivery tasks for cargo without special requirements.

### Limitations of the study

From the perspective of microrobotics, the main contribution of this article is that the proposed capsule microrobots solve the problem of long-range lossless transportation of microrobot swarms. During navigation in flowing media and on 3D uneven surfaces, it is almost impossible to achieve lossless transport of microrobot swarms. Among previous studies, several individual microrobots typically stick to the tissue or are lost in body environments, leading to potential danger.^29^^,^^30^^,^^33^^,^^34^ By leveraging hydrogel membranes to enclose those micro-individuals, the individual loss is significantly reduced to almost zero in the experiments. However, limited by the fabrication process, the physical sizes of the capsule microrobots are relatively large (approximately 2 mm) and cannot navigate crowded vessels. Essentially, this novel design serves as a preorganized strategy for reducing the loss of microrobot groups and can be programmed into a microrobot swarm. Introducing a hydrogel-based production process enables flexible shape designs and efficiently combines microrobot swarms and desired cargos, which is promising for microrobot swarm biomedical applications.

## STAR★Methods

### Key resources table

**Table:** 

REAGENT or RESOURCE	SOURCE	IDENTIFIER
**Chemicals, peptides, and recombinant proteins**		

Sodium alginate ((C_6_H_7_NaO_6_)_n_, 99.5%)	Sinopharm Chemical Reagent Co., Ltd, China.	CAS: 9005-38-3
Calcium chloride (CaCl_2_, 99.7%)	Sinopharm Chemical Reagent Co., Ltd, China.	CAS: 7440-70-2
Fe_3_O_4_	Andy Metal Materials Co. Ltd.	CAS: 1317-61-9

### Resource availability

#### Lead contact

Further information and requests for resources and reagents should be directed to and will be fulfilled by the lead contact, Qingsong Xu (qsxu@um.edu.mo).

#### Materials availability

This study did not generate new unique materials.

### Experimental model and subject details

#### Experimental setup

For electromagnetic actuation experiments, a custom-built five-coil electromagnetic device was fabricated under an optical microscope (SZ61, Olympus Inc., Japan). The five electromagnetic coils were designed to generate a 10 mT-level magnetic field (Figure S5) and were controlled through a real-time controller (NI-9732, National Instrument Ltd., USA). In addition, several NdFeB permanent magnets were utilized to disrupt the capsule microrobot and guide the microrobots (Figure S6). In HEK293 cells experiments, the concentrations of utilized material suspensions were all 1%, where cells were co-cultured for 1 h. Approval of all ethical and experimental procedures and protocols was granted by the Research Ethics Committee of the University of Macau under Application No. APP-ARE-057 and performed in line with the Animal Protection Act enacted by the Legislative Council of Macao Special Administrative Region under Article 71(1) of the Basic Law.

### Methods details

#### Materials preparation

Sodium alginate ((C_6_H_7_NaO_6_)_n_, 99.5%) and calcium chloride (CaCl_2_, 99.7%) were purchased from Sinopharm Chemical Reagent Co., Ltd, China. Calcium hydroxide (Ca(OH)_2_, 95%) was purchased from Tianjin Bodi Chemical Co., Ltd., China. Phenolphthalein solution (0.5%) was obtained from Shandong Linyi Yongan Laboratory, China. Two types of paramagnetic microparticles were utilized, including 3000-mesh Fe_3_O_4_ nanoparticles (<5 μm) and 400-mesh NdFeB (<38 μm) microparticles **(**Figure S1**)**. The chemicals were not further purified in experiments. Microparticles were not further decorated during the entire procedure.

### Quantification and statistical analysis

The error bars represent the extremums. Without other specifications, all experiments were independently repeated at least three times.

## Data Availability

This paper does not report original code. Any additional information required to reanalyze the data reported in this paper is available from the lead contact upon request.
