# Supplementary material for: Versatile magnetic hydrogel soft capsule microrobots for targeted delivery
Source: iScience. 2023 Apr 25;26(5):106727. doi: 10.1016/j.isci.2023.106727 (PMC10192936; doi:10.1016/j.isci.2023.106727)
Supplement: Document S1. Figures S1–S6 [file mmc1.pdf]

iScience, Volume *26*

Supplemental information

Versatile magnetic hydrogel soft capsule microrobots for targeted delivery

Zichen Xu, Zehao Wu, Mingzhe Yuan, Yuanhe Chen, Wei Ge, and Qingsong Xu

Supplemental Figures

Figure S1. Testing and characterization results of the magnetic microparticle, Related to Figure 2.

Figure S2. The disintegration of the capsule microrobot composed of NdFeB microparticles, Related to Figure 2.

Figure S3. Structure stability test result of capsule microrobots in an acidic environment and an alkaline environment, Related to Figure 6.

Figure S4. Comparison of actual images between the control group and experimental groups, Related to Figure 8.

Figure S5. Electromagnetic actuation platform and corresponding simulation results with COMSOL, Related to Figure 8.

Figure S6. Image of the adopted NdFeB permanent magnets and simulation results with COMSOL, Related to Figure 8.

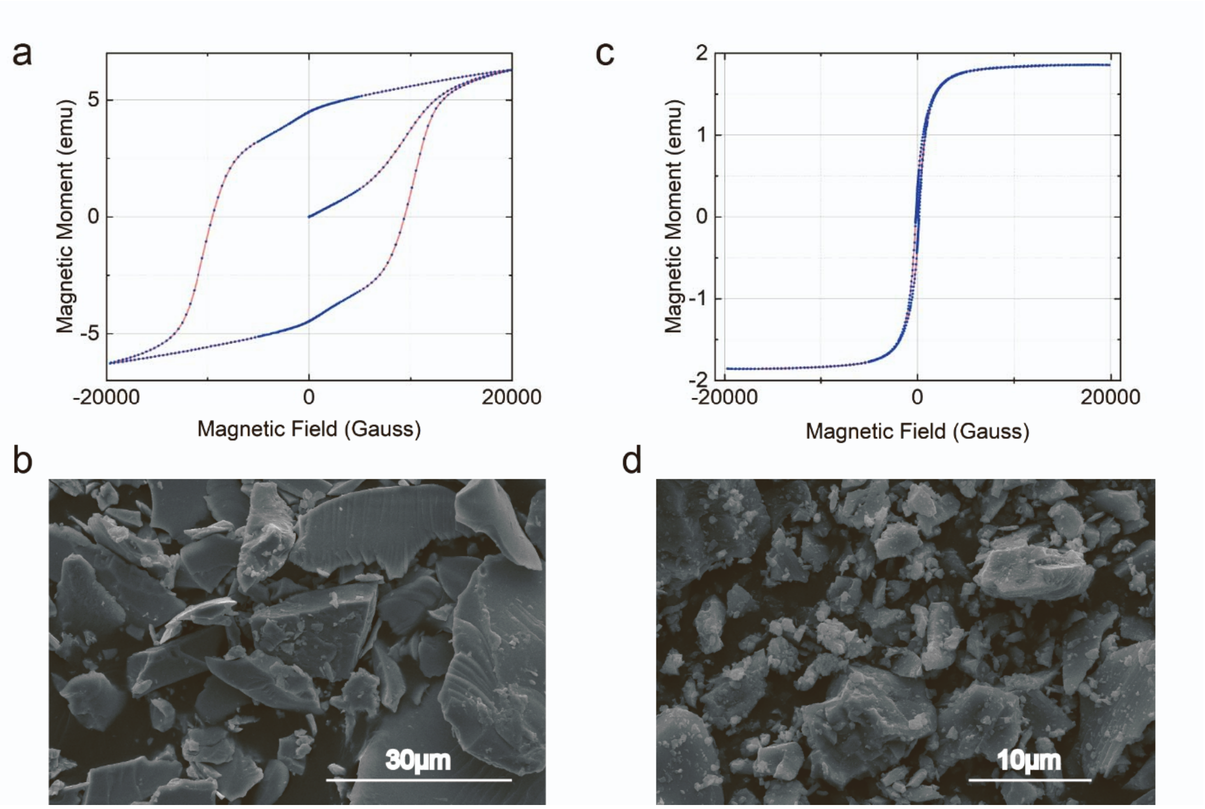

Figure S1. Testing and characterization results of magnetic microparticles, Related to Figure 2. a) The magnetic hysteresis loop of NdFeB microparticles, indicating their magnetization properties. The initial magnetization curves are also provided. b) SEM image of NdFeB microparticles (size <38 μm) shows the microstructures. c) The magnetic hysteresis loop of Fe_3O_4 microparticles, indicating their magnetization properties. The initial magnetization curves are also provided. d) SEM image of Fe_3O_4 microparticles (size <5 μm) shows the microstructures.

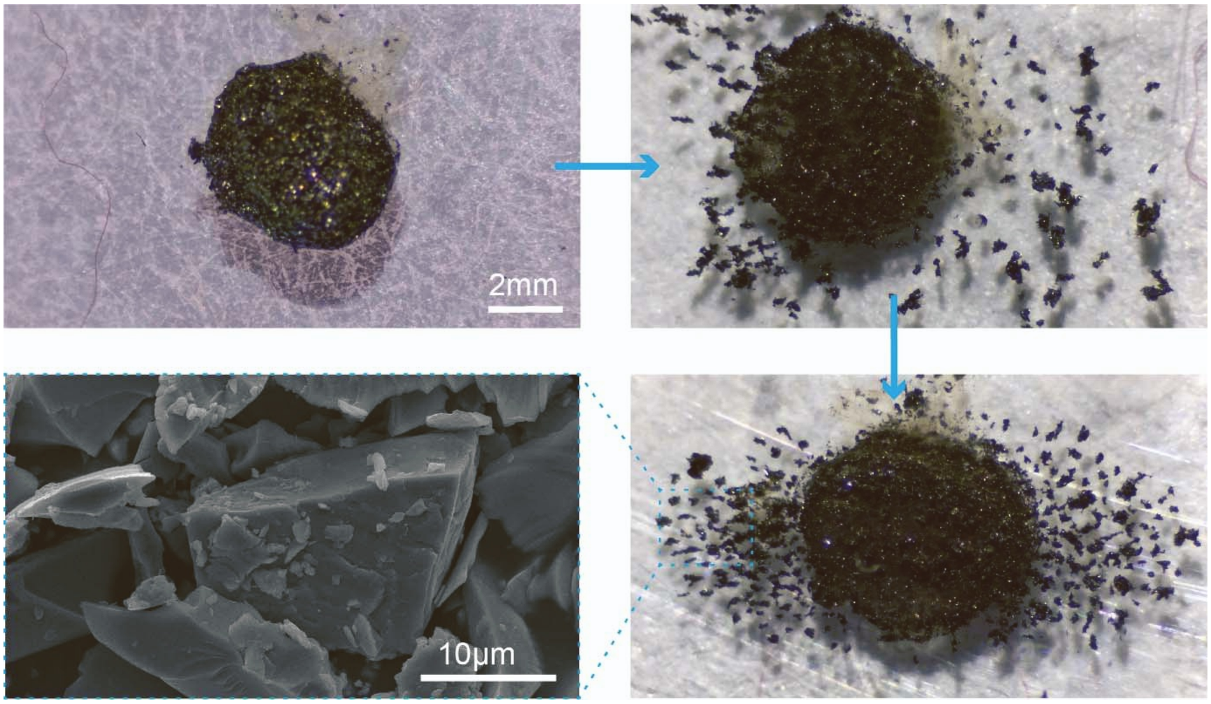

Figure S2. The disintegration of a capsule microrobot composed of NdFeB microparticles (size <5 μm), Related to Figure 2. The size of microparticle is less than 38 μm.

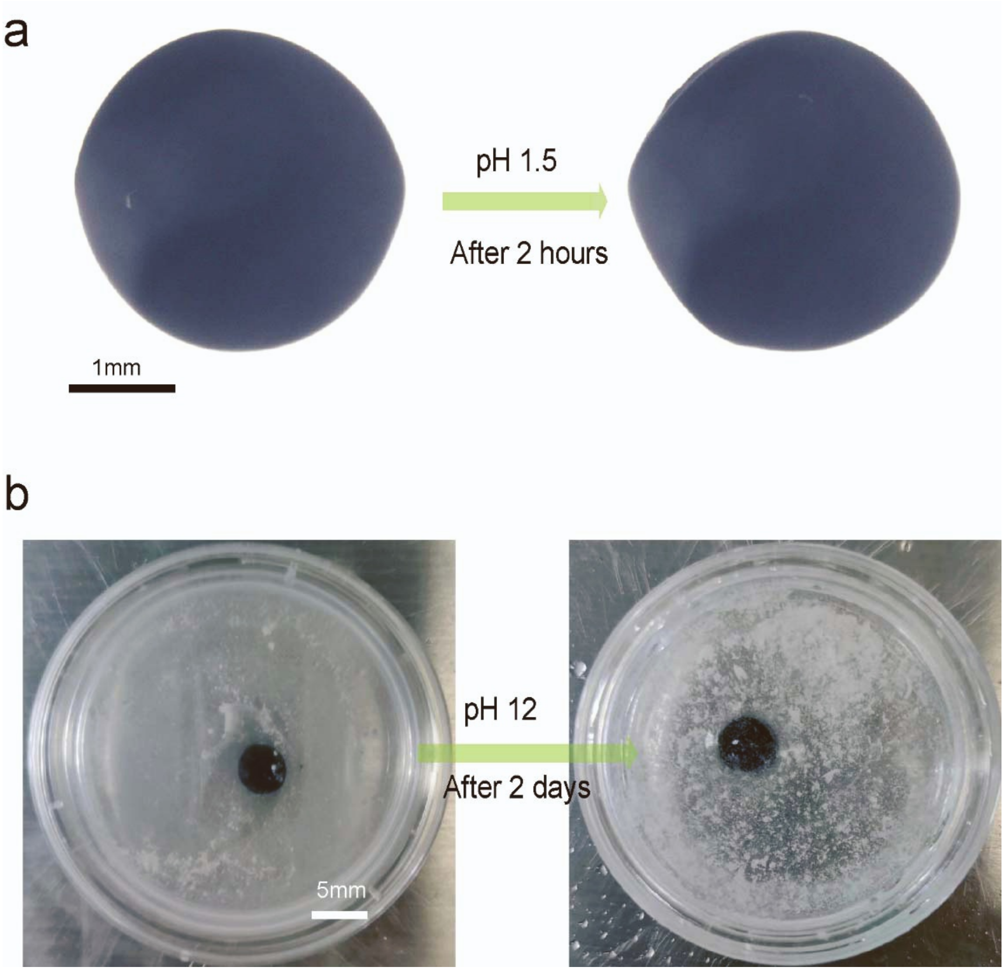

Figure S3. Structure stability test result of capsule microrobots in an acidic environment and an alkaline environment, Related to Figure 6. a) The capsule microrobot can stay stable in an acidic environment (pH 1.5) for more than 2 hours. b) The capsule microrobot can stay stable in an alkaline environment (pH 12) for more than two days. Acidic fluid environments are formed using the HCl solution, and alkaline environments are created by adopting the $Ca(OH)_2$ solution.

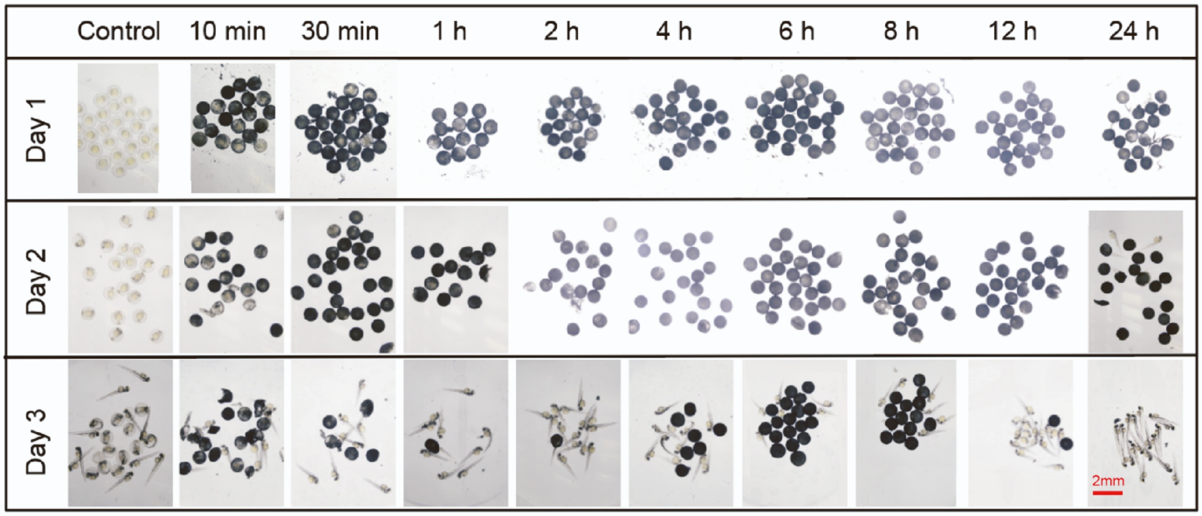

Figure S4. Comparison of actual images between the control group and experimental groups, Related to Figure 8. Nine experimental groups of zebrafish embryos were stored in capsule microrobots for different time intervals and then released into the normal culture. After three days, the majority of zebrafish embryos successfully developed normal morphology.

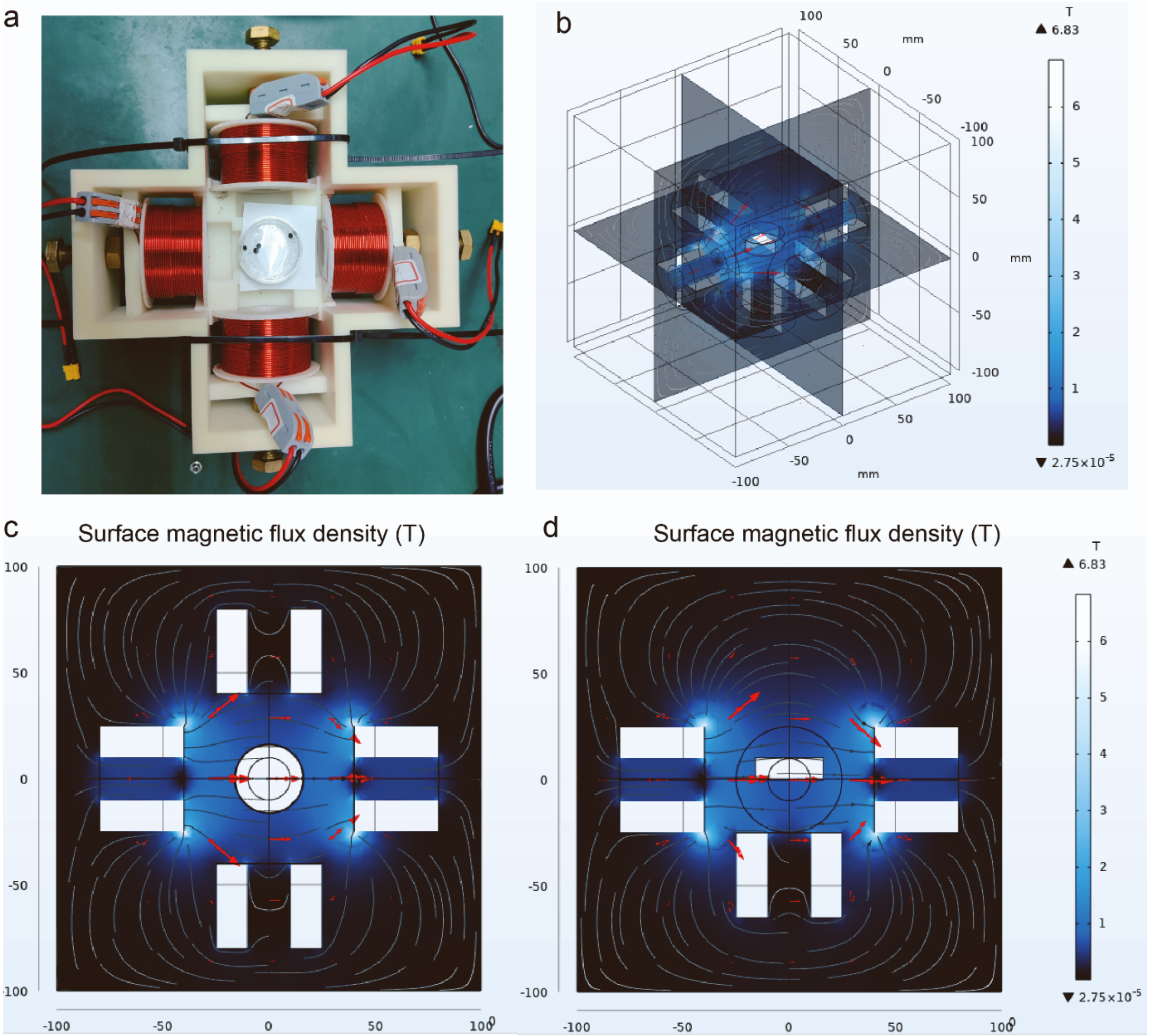

Figure S5. Electromagnetic actuation platform and corresponding simulation results with COMSOL, Related to Figure 8. a) Photo of the electromagnetic actuation platform. b)-d) COMSOL simulation results, including 3-dimensional, top, and side views.

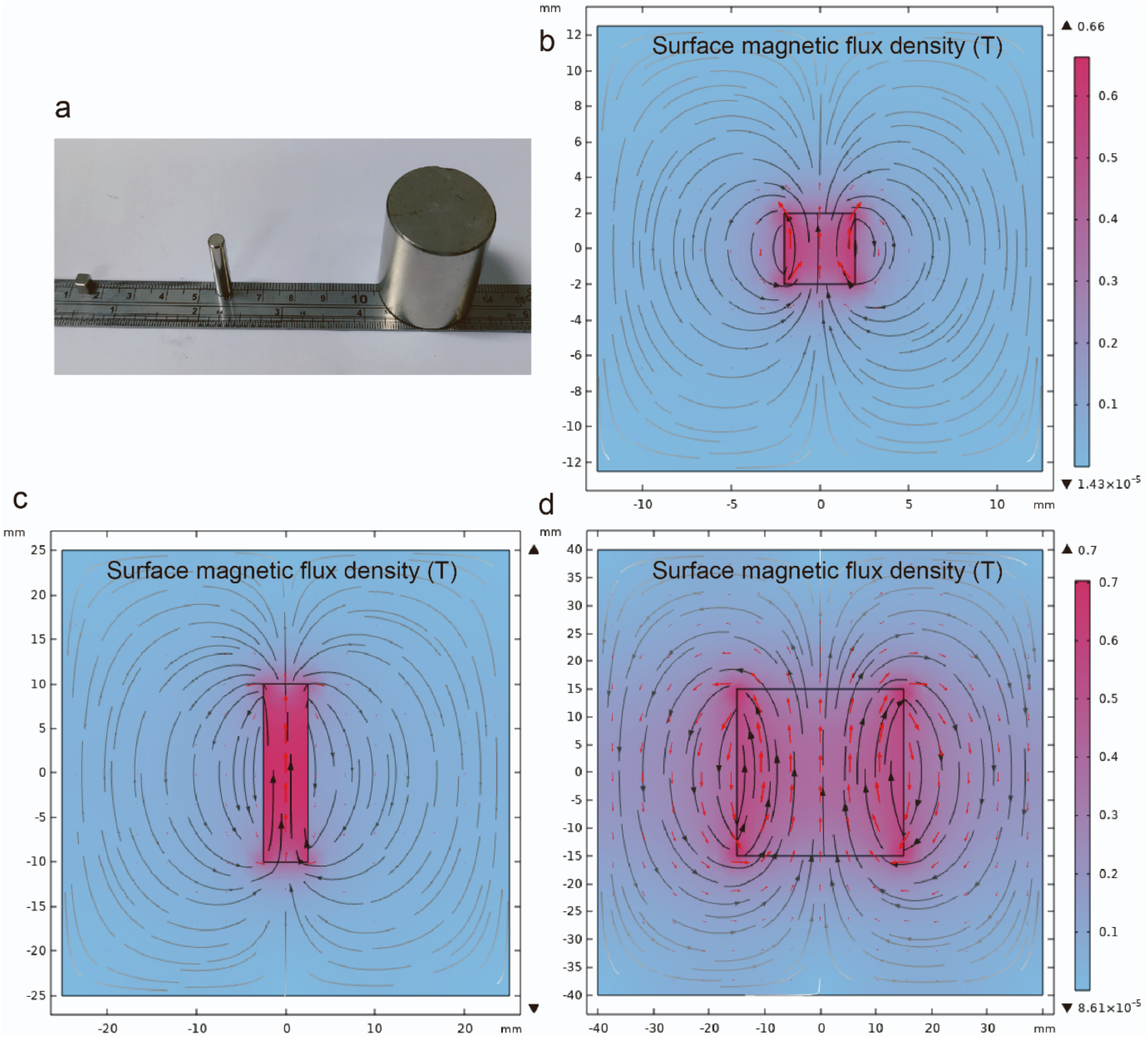

Figure S6. Image of the adopted NdFeB permanent magnets and simulation results with COMSOL, Related to Figure 8. a) Actual images of NdFeB permanent magnets arranged from small to large ones. b)-d) Corresponding simulation results of small and large magnets, respectively.
